# The Effect of Posaconazole, Itraconazole and Voriconazole in the Culture Medium on *Aspergillus fumigatus* Triazole Resistance

**DOI:** 10.3390/microorganisms8020285

**Published:** 2020-02-19

**Authors:** Martyna Mroczyńska, Ewelina Kurzyk, Magdalena Śliwka-Kaszyńska, Urszula Nawrot, Marta Adamik, Anna Brillowska-Dąbrowska

**Affiliations:** 1 Department of Molecular Biotechnology and Microbiology, Gdańsk University of Technology, Narutowicza 11/12, 80-233 Gdańsk, Poland; martyna.mroczynska@pg.edu.pl (M.M.); ewelinakurzyk@gmail.com (E.K.); marta.adamik@gmail.com (M.A.); 2 Department of Organic Chemistry, Gdańsk University of Technology, Narutowicza 11/12, 80-233 Gdańsk, Poland; magdalena.sliwka-kaszynska@pg.edu.pl; 3 Department of Pharmaceutical Microbiology and Parasitology, Faculty of Pharmacy, Wroclaw Medical University, Borowska 213, 50-556 Wroclaw, Poland; urszula.nawrot@umed.wroc.pl

**Keywords:** *Aspergillus fumigatus*, mechanism of azole resistance, azoles

## Abstract

Triazoles are the only compounds used as antibiotics in both medicine and agriculture. The presence of triazoles in the environment can contribute to the acquisition of azole resistance among isolates of *Aspergillus fumigatus*. The objective of this study was to investigate the effect of *A.* *fumigatus* exposure to triazoles on susceptibility to these compounds. Seventeen triazole-resistant and 21 triazole-sensitive *A.* *fumigatus* isolates were examined. The isolates were transferred 20 times on the Sabouraud medium supplemented with posaconazole, itraconazole or voriconazole, followed by five times on the medium not supplemented. The minimum inhibitory concentrations of antimycotics were examined according to the EUCAST broth microdilution method after the 20th transfer and also the 25th transfer. In addition, the expression levels of genes *mdr1*, *mdr2*, *mdr3*, *atrF*, *cyp51*A and *cyp51*B were determined. Cultivation of *A. fumigatus* on media supplemented with posaconazole, itraconazole and voriconazole resulted in the acquisition of resistance to the tested triazoles of all examined isolates. After recultivation on Sabouraud without azoles, most of the isolates lost their acquired resistance. The long-term use of triazole compounds in agriculture may result in the occurrence of triazole resistant *A. fumigatus* isolates in the environment, not only by induction or selection of mutations in the *cyp51*A gene, but also by contribution to changes in the gene expression.

## 1. Introduction

*Aspergillus fumigatus* is a saprophytic mold widespread in the environment. It lives among dead and decaying organic matter in the soil and plays an important role in carbon and nitrogen biogeochemical cycles [1]. In humans, *A. fumigatus* is an opportunistic microorganism that threatens immunocompromised patients. It is the most common clinical cause of a group of illnesses collectively called aspergillosis. These diseases manifest as allergy, colonization or invasive infection [2]. *A. fumigatus* primarily infects the lungs, but can also infect the ears, eyes, skin, mucous membranes and various systemic sites, e.g., urinary tract [3,4]. Invasive aspergillosis is the most serious form of aspergillosis, and has a mortality rate of up to 80% [3]. The estimated burden of disease is about 500,000 acute infections every year [5]. Additionally, 3 million patients suffer from chronic pulmonary aspergillosis [5], and over 4 million from allergic bronchopulmonary aspergillosis [6]. The mortality rate caused by *A. fumigatus* ranges from 30% to 90% in patients with the highest risk (e.g., those immunocompromised by HIV/AIDS) [5,7,8,9].

Triazoles—itraconazole (ITR), voriconazole (VOR), posaconazole (POS) and isavuconazole (ISA)—are the first-line drugs used in aspergillosis therapy however, only VOR, POS and ISA have real-world clinical utility in these infections [10]. In addition to their application in clinical practice, triazoles are also used as fungicides in agriculture [11], as antimycotic agents in material protection and as antifungal drugs for veterinary purposes [12]. Azoles are annually sprayed over plantations of cereals, decorative plants, fruits and vegetables [13]. Their application ranges from the preharvest stage to support growing plants, to the postharvest phase to prevent yield spoilage, and acute treatment of already infected plants. Around 10 mg of azole antifungal is applied to 1 m^2^ of the plant surface area [13]. After their application in households, on fields and in farms, azole residues may enter wastewater treatment plants together with runoff. This phenomenon has been highlighted by research conducted in Spain, Sweden and China, which confirmed the presence of triazoles in sewage sludge [14]. Importantly, sewage sludge is often used as a fertilizer in agriculture and may further contribute to azole persistence in the environment [14]. 

Azole persistence through the environment is alarming. It is estimated that the half-life of azole in the environment is usually over a year [15]. In 2001, it was suggested for the first time that the *A. fumigatus* resistance to medically used azoles may be associated with the use of azole fungicides in agriculture [16]. The hypothesis that the environmental pathway for acquiring resistance may be even more important than the clinical pathway and was strongly supported by Verweij et al. in an opinion piece published in 2009 [17]. As a consequence of these mechanisms, multiazole resistance has been observed even in patients that had never been treated with azoles [18].

From a molecular perspective, azole resistance mechanisms usually involve mutations of the target site in the 14-α sterol demethylase (*cyp51*) gene, up-regulation of the target gene (*cyp51*), overexpression of efflux transporters or stress adaptation by development of a bypass pathway [3]. In the case of *A. fumigatus* isolates, the most important resistance mechanism is comprised of two genetic changes; the first decreases the affinity of the drug for its target (via mutations in the *cyp51*A gene—L98H, Y121F and T289A) and the second causes up-regulation of the synthesis of the target (a 34/46-base pair tandem repeat in the promoter region of the *cyp51*A gene) [19]. The presence of itraconazole in the environment at subinhibitory concentrations that do not inhibit fungal growth results in overexpression of efflux pumps [20]. Multidrug resistance transporters involving the major facilitator superfamily (MFS) and ATP-binding cassette (ABC) transporters lead to elimination of a drug from fungal cells [3,21].

The aim of this research was to investigate whether the azoles present in culture media (posaconazole, itraconazole or voriconazole) contribute to persistent or transient resistance development in *A. fumigatus*, and how they influence the expression level of ABC transporters: *mdr1* (multi-drug resistance protein 1), *mdr2* (multidrug resistance protein 2), *mdr3* (multidrug resistance protein 3), *atrF* (ABC drug exporter) and 14-α sterol demethylases encoded by two separate genes: *cyp51*A and *cyp51*B genes. 

## 2. Materials and Methods 

### 2.1. A. fumigatus Isolates

In this study we examined thirty-eight *A. fumigatus* isolates including 22 clinical isolates (3 posaconazole (POS), itraconazole (ITR), voriconazole (VOR) resistant; 2 POS, ITR resistant and 17 triazole susceptible) and 16 environmental isolates (13 from goose breeding: 11 ITR resistant, 2 triazole susceptible and 3 isolates from air samples collected in countryside: one azole resistant and two azole susceptible). The minimal inhibitory concentration (MIC) value of isolates that are described in Table 1 are listed in Tables S1–S4 (Supplementary Materials). Some of these isolates have been described previously [22,23]. 

### 2.2. Azole Exposure

**Figure 1:**
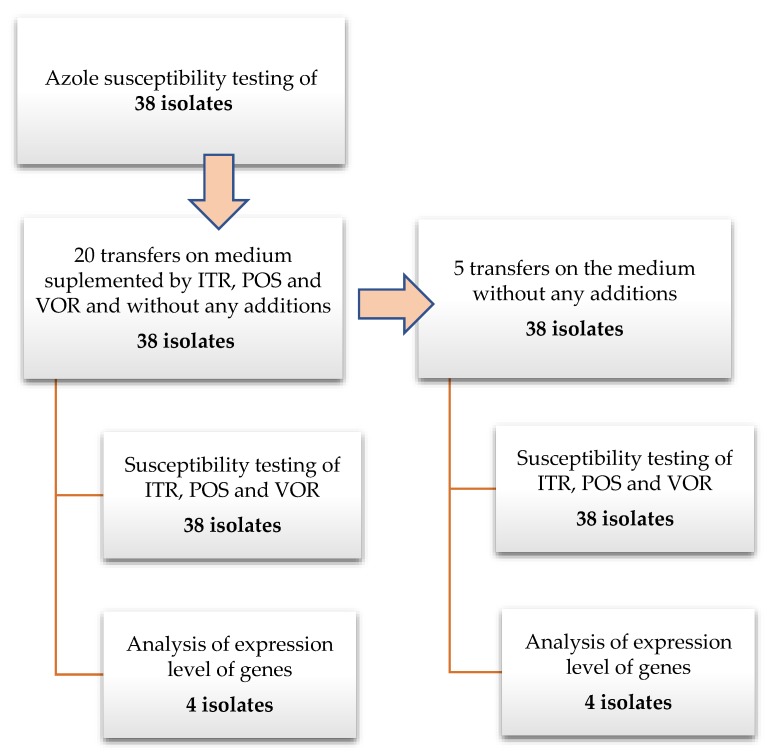
Schema of the experimental protocol.

All studied *A. fumigatus* isolates were transferred twenty times on Sabouraud (SAB) agar plates supplemented separately with triazoles (concentration of individual azoles: ITR—1 mg/L, POS—0.125 mg/L and VOR—0.25 mg/L) and without an antibiotic as a control. The concentration of azoles in the medium has been chosen according to examined isolates MIC values to not reach the inhibitory concentration. The strains were transferred an additional five times on SAB without the addition of triazoles. The isolates were grown at 35 °C during 7 days before the next passage. This time was chosen to allow isolates to produce spores, which were used for antifungal susceptibility testing. After the first and twentieth transfer, and after the fifth transfer on medium without antimycotic supplementation, MIC values were examined. A diagram presenting the performed experiments is presented on Figure 1. To exclude the influence of transferring isolates on the MIC value and/or expressions levels of examined genes, 25 transfers were performed also on SAB without any antimycotics. The antimycotics were purchased in Sigma, German (VOR cat. no. Y0001395; ITR cat. no. I7000000; POS cat. no. 171228-49-2).

### 2.3. Susceptibility Testing

Minimal inhibitory concentration (MIC) was determined for ITR, VOR and POS using the European Committee on Antimicrobial Susceptibility Testing (EUCAST) broth microdilution methods and analyzed according to their recommended clinical breakpoints. RPMI 1640 (with 2% glucose and MOPS (final concentration 0.165 mol/L), pH 7) was prepared to double strength. The final concentration of inoculum was 1 × 10^5^–2.5 × 10^5^ CFU/mL. MIC values were determined after 48 h incubation at 100% inhibition compared with growth control. Isolates were classified as azole resistant when the MIC values were ≥2 mg/L for ITR and VOR and ≥0.25 mg/L for POS. For quality control, according to the EUCAST recommendation, the *A. fumigatus* ATCC 204305 strain was used. SAB (cat. no. 84086), MOPS (cat. no. M3183) and RPMI 1640 have been purchased in Sigma-Aldrich, German. All antifungals were obtained from Sigma-Aldrich.

### 2.4. Gene Expression Analysis

Two triazole resistant and two triazole susceptible isolates were randomly selected for quantitative analysis of expression of *cyp51*A, *cyp51*B, *mdr1* (Afu5g06070), *mdr2* (Afu4g10000), *mdr3* (Afu3g03500), *atrF* (Afu6g04360) and β-tubulin (Afu7g00250) genes [22,24,25]. The list of primer sequences used in this study is present in Table 2. The temperature and time profile of real-time reactions is presented in Table 3. RNA isolation and cDNA synthesis were performed according to the method developed for *A. fumigatus* [22]. RNA was isolated before the experiment and after the first, 20th and 25th transfer. Examination of expression level was performed by real-time PCR using LightCycler 96 PCR Real-Time System (Roche). Real-time PCR was performed in 20 μL reaction volume containing the following reagents: 10 μL Real-Time 2 X PCR Master Mix SYBR (A&A Biotechnology, Poland, cat. no. 2008-100A), 0.1 μL each primer solution (100 mM), 1 μL of total cDNA sample and distilled water. All reactions were performed in triplicates. Quantitative analysis of the level of expression of the investigated genes was carried out using the Pfaffl method [26]. All expression levels refer to the expression level of isolates, grown on a SAB medium without the addition of antimycotics. The results are presented as graph log_2_ (Pfaffl method results). When the value is equal to 0, the level of expression of the tested gene (after culturing on medium with antimycotics) is the same as the strain grown on SAB without azoles. A higher level means an increase of expression. A score lower than 0 means a decrease in the expression level.

## 3. Results

### 3.1. Exposure of A. fumigatus Cultures to Azoles

**Figure 2:**
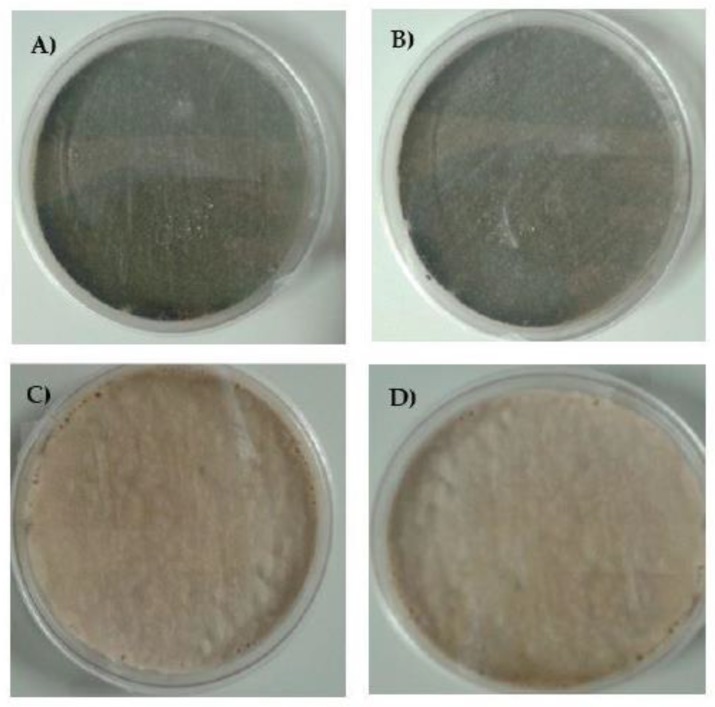
Growth of *A. fumigatus* isolates on (**A**) Sabouraud (SAB), and SAB with addition of (**B**) voriconazole (VOR), (**C**) itraconazole (ITR) and (**D**) posaconazole (POS) after the 1st passage.

*A. fumigatus* classically grows as greenish or dark green-brown colonies. In this experiment, isolates were cultured on medium individually supplemented by azoles at the following concentrations: ITR—1 mg/L, POS—0.125 mg/L and VOR—0.25 mg/L. In the case of the *A. fumigatus* culture on Sabouraud (SAB) agar supplemented by ITR or POS, the growth of mycelium differed from the standard cultures described above. At the first passage, the mycelium of all isolates in the early stages of growth was white, and after 3-4 days the mycelium became “creamish”. Moreover, ITR induced strong growth of mycelium into the agar. We did not observe any changes in coloration and mycelium structure in *A. fumigatus* culture supplemented with VOR. The isolates cultivated on VOR or SAB grew in a classical manner but isolates resistant to ITR grew into SAB medium very strongly. The alteration of mycelium on the medium with different additives is shown in Figure 2. After the 2nd passage, the color of the mycelium for all isolates was green. Surprisingly, even after the third transfer on medium supplemented by POS, we also observed the alteration of classical growth. The mycelium of some isolates were white or white-brown-green, were markedly different to isolates grown on SAB medium. This alteration of growth was observed until the 6th passage, following which isolates grew as green mycelium. All results and photographs are presented in Table S5 and Figures S1–S8.

### 3.2. Azoles Susceptibility Testing

Table 4, Table 5 and Table 6 show the effects of *A. fumigatus* exposure to subinhibitory concentrations of ITR, POS and VOR on the minimum inhibitory concentration (MIC) values for each agent. After 20 transfers (one transfer per week) of isolates on SAB supplemented with ITR and POS, the MIC value for all isolates increased to the highest value tested (>16 mg/L for VOR, and >8 mg/L for ITR and POS). After 20 exposures to VOR, the MIC value of ITR, POS and VOR for most of isolates also increased to the highest tested values. After five transfers on the agar medium without supplementations of azole, the MIC value of all azoles decreased, but not every isolate reached their primary MIC value of ITR, VOR and POS. After 20 transfers on SAB, the results of POS and VOR MIC values were exactly the same. The MIC values of ITR after 20 transfers on SAB were the same or even lower than the initial MIC value (21 were the same and 17 were lower than the initial MIC value). Twenty five transfers on SAB did not influence the MIC value in comparison to the MIC value after 20 transfers on SAB. The results of MIC values examinations for all isolates are presented in Tables S1–S4.

### 3.3. Changes in Gene Expression in Response to Azole Exposure

**Figure 3:**
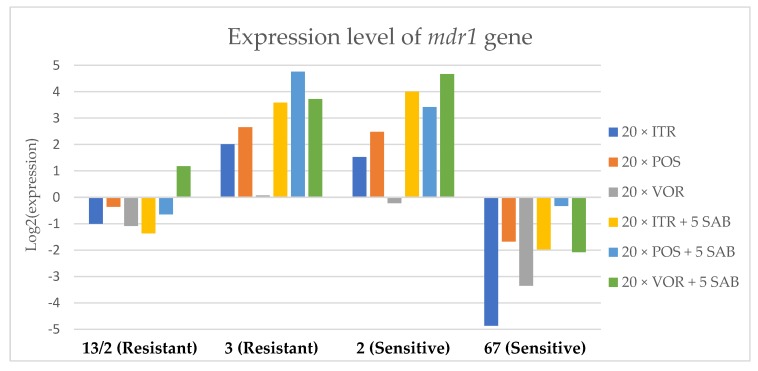
Expression levels of *mdr1* after culturing isolates on SAB with ITR, POS and VOR additions, followed by 5 times culturing on only SAB.

**Figure 4:**
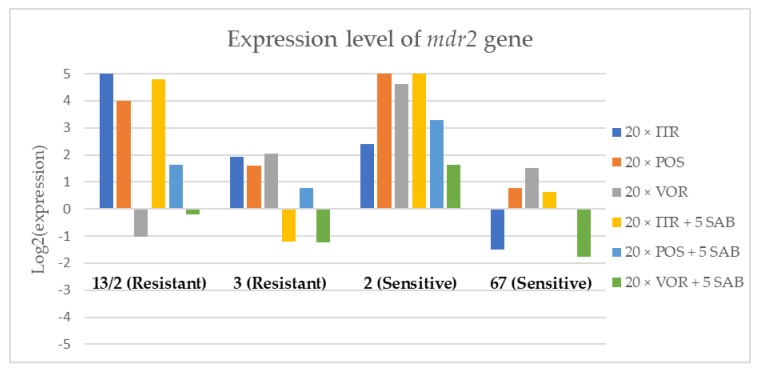
Expression levels of mdr2 after culturing isolates on SAB with ITR, POS and VOR additions, followed by 5 times culturing on only SAB.

**Figure 5:**
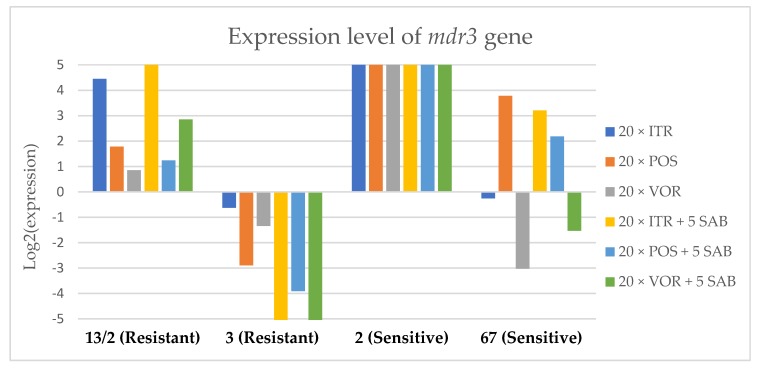
Expression levels of *mdr3* after culturing isolates on SAB with ITR, POS and VOR additions, followed by 5 times culturing on only SAB.

**Figure 6:**
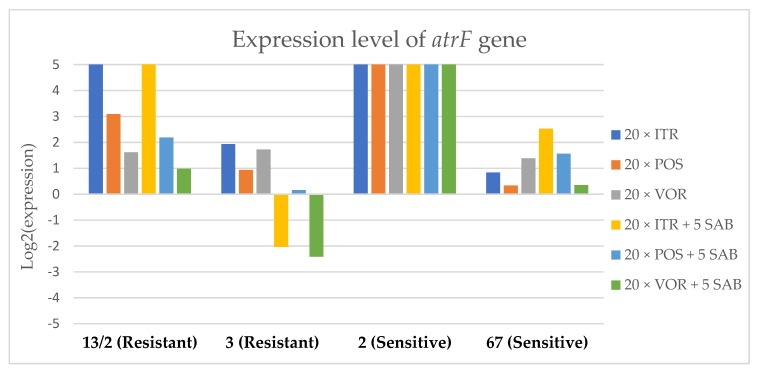
Expression levels of *atrF* after culturing isolates on SAB with ITR, POS and VOR additions, followed by 5 times culturing on only SAB.

**Figure 7:**
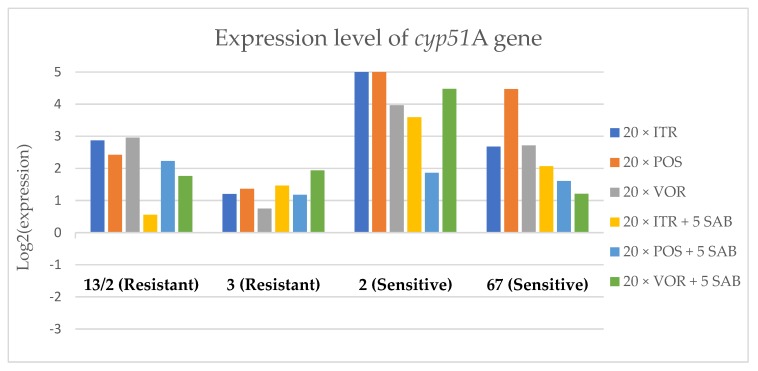
Expression levels of *cyp51*A after culturing isolates on SAB with ITR, POS and VOR additions, followed by 5 times culturing on only SAB.

**Figure 8:**
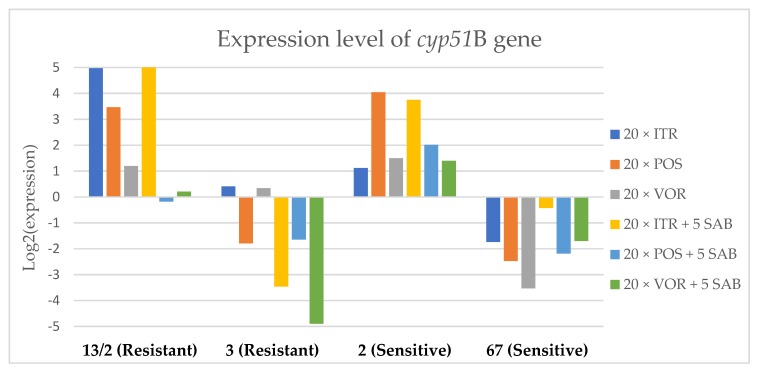
Expression levels of cyp51B after culturing isolates on SAB with ITR, POS and VOR additions, followed by 5 times culturing on only SAB.

To assess the effects if azoles on key gene expression pathways in *A. fumigatus*, we assessed the expression levels of genes encoding the membrane transporters (*mdr1*, *mdr2*, *mdr3* and *atrF*), as well as genes that are involved in the ergosterol biosynthesis pathway of *A. fumigatus* (*cyp51*A and *cyp51*B) after culture in the presence of azoles. Figure 3, Figure 4, Figure 5, Figure 6, Figure 7 and Figure 8 show the expression levels of different genes after culturing isolates on SAB with azole addition, and after five times culturing on only SAB. The means of Cq (threshold cycles) and SD (standard deviation) are listed in the Table S6.

Culture of *A. fumigatus* isolates on SAB supplemented with ITR resulted in a significant increase (up to 5 fold) in the expression levels of *atrF* (Figure 6) genes and *cyp51*A (Figure 7). The expression level of the *mdr1* (Figure 3) gene increased in the case of one resistant isolate (no. 3) and one sensitive isolate (no. 2), whereas the level of expression of the other isolates decreased. *Mdr2* (Figure 4) and *mdr3* (Figure 5) expression levels increased almost five times in the case of two isolates (one sensitive no. 2 and one resistant no. 13/2). *Mdr1*, *mdr2*, *mdr3* and *cyp51*B genes (Figure 3, Figure 4 and Figure 5 and Figure 8) were downregulated in the sensitive isolate no. 67 after culturing on SAB with ITR addition. The expression level of the *cyp51*B (Figure 8) gene was five times upregulated in the resistant isolate 13/2, and in isolates no. 2 and 3, the *cyp51*B expression level increased or remained as the initial expression. As a result of retransfer of isolates from SAB with ITR onto SAB without azole supplementation, gene expression decreased but did not return to initial levels. The expression of *mdr2*, *mdr3*, *atrF* and *cyp51*B was downregulated only in resistant isolate no. 3 after retransfers on SAB medium, whereas the expression levels of *mdr1* and *cyp51*A were higher than their expression after 20 transfers on SAB with ITR addition.

Culturing the isolates on SAB with POS resulted in a significant increase (3-5 fold higher) in the expression level of *mdr2*, *atrF* and *cyp51*A genes in all tested isolates (Figure 4, Figure 6 and Figure 7). The change in expression levels of *mdr1*, *mdr3* and *cyp51*B depended on the strains tested. After culture on SAB supplemented with POS, the expression level of the all tested genes followed the same trend; if some genes were downregulated they remained downregulated. Comparing to the initial gene expression levels, the upregulation of gene expression in isolates after five retransfers on SAB without supplementation was lower than after cultivation on SAB supplemented with POS medium. 

Culture of the isolates on SAB with VOR (Figure 3, Figure 4, Figure 5, Figure 6, Figure 7 and Figure 8) resulted in upregulation of the *cyp51*A and *atrF* genes in all tested isolates. The expression of *mdr1* after exposure to VOR is downregulated for all isolates. As a result of retransfer on SAB after VOR exposure, the expression level of the *cyp51*A remained upregulated. Genes *cyp51*B, *mdr2* and *mdr3* were downregulated for three isolates except sensitive isolate no. 2. The expression level of *mdr1* increased up to 5-fold after removal of VOR addition. This is the only isolate in which we found downregulation after VOR addition and upregulation after removal of the VOR.

To obtain a better understanding of gene expression patterns, the gene expression of individual isolates was also considered. The clinical isolate no. 13/2 was characterized by TR_34_/L98H mutation in the *cyp51*A gene, and was resistant to ITR, POS and VOR. The expression levels of *mdr2*, *mdr3*, *atrF*, *cyp51*A and *cyp51*B increased after 20 times exposure to all tested azoles (with one exception, after VOR exposition the *mdr2* gene was downregulated). However, the expression level of the *mdr1* gene for this strain was downregulated after 20 times exposure on all three tested azoles. After 20 times exposure to POS or VOR and five passages only on SAB, the expression levels were decreased, but were still higher than the initial level. After 20 times exposure to ITR and five SAB transfers, the expression level of tested genes were the same as after exposition to ITR, only the expression of *cyp51*A was lower than the expression level after 20 times ITR exposition.

The environmental isolate no. 3 was also characterized by a TR_34_/L98H mutation in the *cyp51*A gene, and was resistant to ITR, POS and VOR. The expression levels of the genes *mdr1*, *mdr2*, *atrF* and *cyp51*A were upregulated after 20 times of azole exposure. After the removal of azoles from the medium and reculturing this isolate on a SAB only medium, the expression of *mdr1* was the same, *cyp51*A expression was even higher than after azole exposure. The expression of *mdr2*, *mdr3*, *atrF* and *cyp51*B was downregulated after removing the azoles addition from the medium. After exposure to azoles, and also after the removal of azoles, the genes *mdr3* and *cyp51*B were downregulated or remained at the initial levels. The higher expression level of *cyp51*B was identified in isolates without mutation in *cyp51*A after exposure on 1 mg/L of ITR [27].

The clinical isolate no. 2 was sensitive to ITR, POS and VOR, and had no mutation in the *cyp51*A gene. The expression levels of all six genes were increased after azole exposure, and this higher level also remained after the removal of azole and cultured on SAB. The expression levels of many tested genes in this isolate increased 5-fold compared to the initial expression level of those genes. Moreover, the expression levels reached the highest value among all tested isolates.

The isolate no. 67 was sensitive to tested azoles, had no mutation in gene *cyp51*A and was isolated from goose. This isolate was characterized by downregulation of *mdr1* and *cyp51*B genes after exposure to ITR, POS and VOR. Upregulation was observed for *cyp51*A and *atr*F after azole exposure and following five times transfers on SAB. The expression level of *mdr2* and *mdr3* were differentially regulated, depending on the azole the isolate was exposed to. 

Summing up the gene expression patterns in isolates grown with the addition of different azoles, we found that all of the studied azoles (POS, VOR and ITR) caused a significant increase in expression of the *cyp51*A and *atrF* genes of all tested *A. fumigatus* isolates. We also observed that POS induced an increase in the expression level of the *mdr2* gene in all tested isolates. Removal of POS from the medium produced the same expression level, and in some cases contributed to higher expression, of the examined genes. However, removal of the ITR from the environment resulted in upregulation of the examined genes, or retained the expression level observed after ITR exposure in the majority of isolates. Removal of VOR from the environment contributed to a lower expression level of *mdr2* (Figure 4). 

## 4. Discussion

Our research was undertaken to examine and establish how the presence of POS, ITR and VOR in the environment influences azole resistance development in *A. fumigatus*. Performing a resistance induction trial on susceptible Polish environmental strains was a novel approach, involving creating environmental pressure to stimulate adaptation of fungi to the presence of therapeutic azoles. The obtained results confirmed that the use of medically applied azoles can induce changes in resistance of clinical and environmental, azole susceptible and resistant *A. fumigatus* isolates, and can influence the gene expression of transporters. Based on our results, it is clear that azole pressure caused acquired resistance in susceptible environmental strains. The examined strains grew on medium supplemented by azole and produced spores. All isolates not only survived during 20 passages on medium with addition of azoles, but grew very abundantly. The research outcomes of our work reflect the findings of past studies. It has been previously shown that azole fungicides present in culture medium can provoke cross-resistance to medical azole drugs in *A. fumigatus* after only three transfers [11]. Another series of weekly transfers on azole fungicides at different concentrations was also able to cause resistance. Consistent with our findings, a significant MIC increase was noted in all examined strains. For instance, an isolate with initial MIC of 0.25 mg/L for ITR, 0.015 mg/L for POS and 0.25 mg/L for VOR, had MICs equal to 2 mg/L, 1 mg/L and 16 mg/L, respectively, after culturing on tebuconazole [28]. The MIC values of the isolates presented in this study were significantly higher than the MIC values obtained by other research teams [28]. This could be the result of higher number of transfers of the tested isolates, and as a consequence, longer exposure times of the azole compounds.

The relationship between the presence of the TR_34_/L98H and TR_46_/Y121F/T289A mutations and resistance to azoles is well documented [18,19,20,21]. In this work, we reported differences in the expression of *mdr1, mdr2, mdr3, atrF, cyp51*A and *cyp51*B genes of the *A. fumigatus* when grown on SAB and SAB supplemented with VOR, POS or ITR (20 transfers on these substrates adequately) and five retransfers on SAB without azoles. The sensitive isolates were characterized by higher expression level of all examined genes than the resistance isolates. This is likely the reason why those sensitive isolates not only survived, but also grew on the medium supplemented by higher addition of azoles. Gene expression of resistant isolates was different; when one gene was upregulated in clinical isolate no. 13/2, the same gene was downregulated in environmental isolates no. 3. Only expression of *cyp51*A was upregulated in both isolates. This may suggest that those isolates acquired resistance through different mechanisms, especially as one was isolated from the patient, and another from the environment. Perhaps exposure to agriculture azole contributes only to acquired mutations in the *cyp51*A gene, but not to alterations in gene expression. This hypothesis should be further tested in future work.

The higher expression level of examined genes after removal of azoles compared to the initial level appears counterintuitive. However, the upregulation of efflux transporter gene expression is not a consequence of resistance among sensitive isolates, it can only be an effect of influence of azoles on metabolism [29].

Interestingly, the expression level of some genes was lower than the base level of expression. According to the research Fraczek et al., the expression level of *mdr1*, *mdr2* and *mdr3* during normal growth at sensitive strains are lower than 20% of β-tubulin expression [30,31,32,33].

The MIC value for the four isolates after exposure to three azole compounds was higher than the initial MIC values. However, after five retransfers on the SAB medium, the MIC values came back to the initial level (with one exception, the MIC values of isolate no. 67 were higher than the initial but lower than after azole exposure; Tables S1–S4). 

The majority of environmental isolates from geese breeding were initially only resistant to ITR, but after cultivation on SAB substrate, lost resistance to ITR. Perhaps these isolates were exposed to azoles in the environment, which resulted in changes in genes expression and in ITR resistance. During experiments in laboratory, after five recultivating rounds on SAB, the isolates lost their resistance. These isolates were characterized by temporal resistance to azoles caused by a different mechanism than the TR34/L98H and TR46/Y121F/T289A mutation in the *cyp51*A gene. The information about genetic background of these isolates is presented in Table 1.

Our research indicate that azole exposition induce acquired mechanism of resistance, especially overexpression of *cyp*51A among both resistant and sensitive isolates. Therefore, the use of azoles in medical practice is associated with the risk of acquiring resistance. In addition, after removal of the drug, and thus the cessation of azoles as a prophylaxis, only in a few cases a reduction in expression of factors responsible for acquired resistance was observed. According to this after azole prophylaxis alternative groups of antimycotics should be used to obtain the best results of antifungal therapy. This hypothesis will be further examined, more clinical and environment isolates will be tested.

## Figures and Tables

**Table 1:** Characterization of *Aspergillus fumigatus* isolates, cyp51 gene mutations and azole susceptibility testing.

No. of Isolate	Place of Isolates Origin	Mutations at Sequence of:		TR_34_	Resistance			Reference
		Nucleotide	Amino Acid		ITR	POS	VOR	
2	**Clinical isolates from Wroclaw**	-	-	-	-	-	-	[22,23]
13/2		T293A	Leu-His	+	+	+	+	[22,23]
25		-	-	-	-	-	-	[23]
29		G784T	Asp-Tyr	-	-	-	-	[23]
55		T293A	Leu-His	+	+	+	+	[22,23]
72		-	-	-	-	-	-	[23]
73		-	-	-	-	-	-	[23]
79		-	-	-	-	-	-	[23]
1	Clinical isolates from Warsaw	T229G G393A G433A	Leu-Val Gln-Gln Glu-Lis	-	-	-	-	[23]
5		T229G	Leu-Val	-	-	-	-	[23]
12		T229G G333C	Leu-Val Thr-Thr	-	-	-	-	[23]
13/3		T229G G313A	Leu-Val Glu-Lys	-	-	-	-	[23]
14		G247T	Val-Phe	-	-	-	-	[23]
15		T293A	Leu-His	+	+	+	-	[23]
16		A217T T229G	Thr-Pro Leu-Val	-	-	-	-	[23]
20		T441G	His-Gln	-	-	-	-	[23]
21		T293A	Leu-His	+	+	+	+	[23]
22		-	-	-	-	-	-	[23]
24		T293A	Leu-His	+	+	+	-	[23]
37A		G393A G433A	Gln-Gln Glu-Lys	-	-	-	-	[23]
132H		T744A T912G	Asp-Lys Trp-Cys	-	-	-	-	[23]
1G	Clinical isolates from Gdansk	A81C	Leu-Phe	-	-	-	-	-
64	Goose breeding	G777C	Gln-His	-	+	-	-	-
67		-	-	-	-	-	-	-
83		T667G C672T	Trp-Gly Ala-Ala	-	+	-	-	-
84		G423A	Gln-Gln	-	+	-	-	-
85		G885T	Gln-His	-	+	-	-	-
104		C672T	Ala-Ala	-	+	-	-	-
103		-	-	-	+	-	-	-
106		-	-	-	-	-	-	-
113		G423A G713A G905A	Gln-Gln Arg-His Ser-Asn	-	+	-	-	-
121		G423A	Gln-Gln	-	+	-	-	-
140		G423A G777C	Gln-Gln Gln-His	-	+	-	-	-
141		G423A	Gln-Gln	-	+	-	-	-
143		-	-	-	+	-	-	-
3	Environmental isolates from Gdansk and surroundings	T293A	Leu-His	+	+	+	+	-
5		T229G	Leu-Val	-	-	-	-	-
8		G393A	Gln-Gln	-	-	-	-	-

* strain no. 13/2 was deposited in CBS KNAW—CBS 133436.

**Table 2:** The list of primers sequence used on this study.

Gene	Primer sequence (5′ –> 3′)	Reference
*mdr1*	GCTCTTCCCTTGTTCACAATTC	This study
	CGGCAATACCGAGATACACA	
*mdr2*	TTTAGCTCCACCGGGTTTG	[25]
	TCGAAAGACCGAACATGCTTGA	
*mdr3*	TCTGATGGCGGTCATCACT	[25]
	ATATCCATCCCCCAGGC	
*atrf*	AGAGAAATCGGACAACTGCTGA	[25]
	CCTCGTCGCAGATAGTCTTGTA	
*cyp51A*	TGCAGAGAAAAGTATGGCGA	[25]
	CGCATTGACATCCTTGAGC	
*cyp51B*	AGCAGAAGAAGTTCGTCAAATAC	[25]
	TCGAAGACGCCCTTGTG	
β-tubulin	TTCCCCCGTCTCCACTTCTTCATG	[26]
	GACGAGATCGTTCATGTTGAACTC	

**Table 3:** Time and temperature profile of all genes tested in real-time PCR.

Steps of Real-Time PCR	Temperature (°C)	Time (s)	Ramp (°C/s)
Initial denaturation	95	300	4.4
Denaturation	95	30	4.4
*cyp*51A, c*yp*51B	95	15	4.4
*mdr*1, m*dr*2, m*dr*3, a*tr*F, *β-tubulin*	95	30	4.4
Annealing			
*cyp*51A	60	15	2.2
*cyp*51B	52	15	2.2
*mdr*1	59	60	2.2
*mdr*2, m*dr*3	62	60	2.2
*atr*F, *β-tubulin*	60	60	2.2
Extension			
*cyp*51A, c*yp*51B	72	20	4.4
*mdr*1, m*dr*2, m*dr*3, a*tr*F, *β-tubulin*	72	60	4.4
Denaturation	95	10	4.4
Melting of PCR products	60	60	2.2
	97	60	0.1
Cooling	40	300	2.2

**Table 4:** The effects of exposure to subinhibitory concentrations of ITR, VOR or POS on the minimal inhibitory concentration (MIC) values for each agent.

MIC Value (mg/L)	Number of Isolates with Defined ITR MIC Values after Exposure Isolates on Respective Agents								
	Initial	20 SAB *	20 SAB + 5 SAB **	20 ITR	20 ITR + 5 SAB	20 VOR	20 VOR + 5 SAB	20 POS	20 POS + 5 SAB
>8	3	1	1	38	3	27	3	38	3
8	8	4	4	0	2	8	3	0	2
4	5	0	0	0	0	3	7	0	0
2	1	1	1	0	6	0	4	0	5
1	1	1	1	0	6	0	1	0	7
0.5	12	16	16	0	11	0	12	0	12
0.25	7	12	12	0	9	0	7	0	8
0.125	1	3	3	0	1	0	1	0	1
0.0625	0	0	0	0	0	0	0	0	0
0.032	0	0	0	0	0	0	0	0	0
0.016	0	0	0	0	0	0	0	0	0

* 20 SAB (ITR) isolates were cultured 20 times on SAB medium without supplementation (on SAB with addition of ITR), ** 20 SAB (ITR) + 5 SAB isolates were cultured 20 times on SAB medium without supplementation (on SAB with addition of ITR), and then were cultured 5 times on SAB medium without supplementation.

**Table 5:** The effects of exposure to subinhibitory concentrations of ITR, VOR or POS on the MIC values for the respective agents.

MIC Value (mg/L)	Number of Isolates with Defined POS MIC Values after Exposure Isolates on Respective Agents								
	Initial	20 SAB	20 SAB + 5 SAB	20 ITR	20 ITR + 5 SAB	20 VOR	20 VOR + 5 SAB	20 POS	20 POS + 5 SAB
>8	0	0	0	38	0	33	0	38	0
8	0	0	0	0	0	4	0	0	0
4	0	0	0	0	0	1	0	0	0
2	0	0	0	0	0	0	2	0	0
1	0	0	0	0	0	0	3	0	0
0.5	3	3	3	0	8	0	7	0	8
0.25	3	3	3	0	5	0	5	0	4
0.125	5	5	5	0	6	0	2	0	5
0.0625	13	13	13	0	9	0	9	0	11
0.032	12	12	12	0	8	0	8	0	8
0.016	2	2	2	0	2	0	2	0	2

**Table 6:** The effects of exposure to subinhibitory concentrations of ITR, VOR or POS on the MIC values for the respective agents.

MIC Value (mg/L)	Number of Isolates with Defined VOR MIC Values after Exposure Isolates on Respective Agents								
	Initial	20 SAB	20 SAB + 5 SAB	20 ITR	20 ITR + 5 SAB	20 VOR	20 VOR + 5 SAB	20 POS	20 POS + 5 SAB
>16	0	0	0	38	0	35	0	38	0
16	0	0	0	0	0	3	0	0	0
8	0	0	0	0	0	0	0	0	0
4	0	0	0	0	0	0	0	0	0
2	4	4	4	0	5	0	4	0	4
1	2	2	2	0	7	0	8	0	4
0.5	5	5	5	0	6	0	6	0	9
0.25	22	22	22	0	19	0	17	0	20
0.125	5	5	5	0	1	0	3	0	1
0.0625	0	0	0	0	0	0	0	0	0
0.032	0	0	0	0	0	0	0	0	0

## Supplementary Materials

The following are available online at https://www.mdpi.com/2076-2607/8/2/285/s1. Table S1: The MIC values of ITR, POS and VOR after 20 and 25 transfers on Sabouraud agar Table S2: The MIC values of ITR, POS and VOR after 20 transfers on Sabouraud agar with addition of ITR, and then five transfers only on Sabouraud agar without any additions. Table S3: The MIC values of ITR, POS and VOR after 20 transfers on Sabouraud agar with addition of POS, and then five transfers only on Sabouraud agar without any additions. Table S4: The MIC values of ITR, POS and VOR after 20 transfers on Sabouraud agar with addition of VOR, and then five transfers only on Sabouraud agar without any additions. Table S5: The alteration of *A. fumigatus* mycelium after exposition of POS, ITR and VOR. Figure S1: The growth of *A. fumigatus* isolate on SAB medium without any additions. Figure S2: Colony morphology of isolate no. 2 cultured on A) SAB, B) SAB supplemented by 0.25 mg/L of VOR. Figure S3: Colony morphology of isolate no. 2 cultured on A) SAB, B) SAB supplemented by 1 mg/L of ITR. Figure S4: Colony morphology of isolate no. 2 cultured on A) SAB, B) SAB supplemented by 0.125 mg/L of POS. Figure S5: Colony morphology of isolate no. 2 cultured on A) SAB, B) SAB supplemented by 1 mg/L of ITR. Figure S7: Colony morphology of isolate no. 3 cultured on SAB supplemented by VOR, ITR, POS and only SAB after 3-5th passages. Figure S8: Colony morphology of isolate no. 55 cultured on SAB supplemented by VOR, ITR, POS and only SAB after 3–5th passages. Table S6: Raw data of cycle quantification values and standard deviation of tested genes.
