# Supplementary material for: The Effect of Posaconazole, Itraconazole and Voriconazole in the Culture Medium on Aspergillus fumigatus Triazole Resistance
Source: Microorganisms. 2020 Feb 19;8(2):285. doi: 10.3390/microorganisms8020285 (PMC7094209; doi:10.3390/microorganisms8020285)
Supplement: Supplementary file 1 [file microorganisms-08-00285-s001.pdf]

microorganisms

MDPI

Supplementary materials:

The results of MIC values investigations

Table S1. The MIC values of ITR, POS and VOR after 20 and 25 transfers on Sabouraud agar.

		MIC value [mg/L] after 24 and 48 hours					
**Tested azoles**	**Place of isolates origin**	**ITR**		**POS**		**VOR**	
**Isolates**		**Initial value**	**After 20/25th**	**Initial value**	**After 20/25th**	**Initial value**	**After 20/25th**
**2**	**Clinical isolates from Wroclaw**	0.25	0.25	0.0312	0.0312	0.25	0.25
**13/2**		2	2	0.5	0.5	2	2
**25**		0.5	0.5	0.0312	0.0312	0.25	0.25
**29**		0.25	0.25	0.0156	0.0156	0.25	0.25
**55**		>8	8	0.25	0.25	2	2
**72**		0.25	0.25	0.0312	0.0312	0.125	0.125
**73**		0.125	0.125	0.0156	0.0156	0.25	0.25
**79**		0.25	0.125	0.0312	0.0312	0.25	0.25
**1**	Clinical isolates from Warsaw	0.5	0.25	0.06	0.06	0.5	0.5
**5**		0.5	0.25	0.03	0.03	0.25	0.25
**12**		0.5	0.5	0.03	0.03	0.25	0.25
**13/3**		0.25	0.25	0.03	0.03	0.25	0.25
**14**		0.5	0.5	0.06	0.06	0.25	0.25
**15**		8	8	0.5	0.5	1	1
**16**		0.5	0.5	0.06	0.06	0.25	0.25
**20**		0.5	0.5	0.03	0.03	0.25	0.25
**21**		>8	8	0.5	0.5	2	2
**22**		1	1	0.06	0.06	0.5	0.5
**24**		8	8	0.25	0.25	1	1
**37A**		0.5	0.5	0.06	0.06	0.25	0.25
**132H**		0.5	0.5	0.06	0.06	0.25	0.25
**1G**	Clinical isolates from Gdansk	0.5	0.5	0.06	0.06	0.25	0.25
**64**	Geese breeding	4	0.25	0.03	0.03	0.5	0.5
**67**		0.5	0.5	0.06	0.06	0.25	0.25
**83**		8	0.5	0.03	0.03	0.25	0.25
**84**		8	0.5	0.03	0.03	0.25	0.25
**58**		4	0.25	0.06	0.06	0.25	0.25
**104**		4	0.25	0.06	0.06	0.125	0.125
**103**		8	0.5	0.125	0.125	0.25	0.25
**106**		0.5	0.5	0.125	0.125	0.25	0.25
**113**		4	0.25	0.03	0.03	0.5	0.5
**121**		8	0.5	0.06	0.06	0.125	0.125
**140**		8	0.5	0.125	0.125	0.125	0.125
**141**		8	0.5	0.125	0.125	0.125	0.125
**143**		4	0.25	0.125	0.125	0.5	0.5
**3**	Environmental isolates from Gdansk and surroundings	>8	>8	0.25	0.25	2	2
**5**		0.25	0.25	0.06	0.06	0.25	0.25
**8**		0.25	0.125	0.06	0.06	0.25	0.25

Table S2. The MIC values of ITR, POS and VOR after 20 transfers on Sabouraud agar with addition of ITR, and then 5 transfers only on Sabouraud agar without any additions.

		MIC values [mg/L] after 24 and 48 hours								
Tested azoles	Place of isolates origin	**ITR**			**POS**			**VOR**		
Isolates		**Initial value**	**After 20th**	**After 25th**	**Initial value**	**After 20th**	**After 25th**	**Initial value**	**20th**	**25th**
2	**Clinical isolates from Wroclaw**	0.25	>8	0.25	0.0312	>8	0.0312	0.25	>16	0.25
13/2		2	>8	2	0.5	>8	0.5	2	>16	2
25		0.5	>8	0.5	0.0312	>8	0.0312	0.25	>16	0.25
29		0.25	>8	0.25	0.0156	>8	0.0156	0.25	>16	0.25
55		>8	>8	>8	0.25	>8	0.25	2	>16	2
72		0.25	>8	0.25	0.0312	>8	0.0312	0.125	>16	0.125
73		0.125	>8	0.125	0.0156	>8	0.0156	0.25	>16	0.25
79		0.25	>8	0.25	0.0312	>8	0.0312	0.25	>16	0.25
1	Clinical isolates from Warsaw	0.5	>8	0.5	0.06	>8	0.06	0.5	>16	0.5
5		0.5	>8	0.5	0.03	>8	0.03	0.25	>16	0.25
12		0.5	>8	0.5	0.03	>8	0.03	0.25	>16	0.25
13/3		0.25	>8	0.25	0.03	>8	0.03	0.25	>16	0.25
14		0.5	>8	0.5	0.06	>8	0.06	0.25	>16	0.25
15		8	>8	8	0.5	>8	0.5	1	>16	1
16		0.5	>8	0.5	0.06	>8	0.06	0.25	>16	0.25
20		0.5	>8	0.5	0.03	>8	0.03	0.25	>16	0.25
21		>8	>8	>8	0.5	>8	0.5	2	>16	2
22		1	>8	1	0.06	>8	0.06	0.5	>16	0.5
24		8	>8	8	0.25	>8	0.25	1	>16	1
37A		0.5	>8	0.5	0.06	>8	0.06	0.25	>16	0.25
132H		0.5	>8	0.5	0.06	>8	0.06	0.25	>16	0.25
1G	Clinical isolates from Gdansk	0.5	>8	0.5	0.06	>8	0.06	0.25	>16	0.25
64	Geese breeding	4	>8	1	0.03	>8	0.125	0.5	>16	2
67		0.5	>8	2	0.06	>8	0.25	0.25	>16	1
83		8	>8	2	0.03	>8	0.125	0.25	>16	1
84		8	>8	2	0.03	>8	0.5	0.25	>16	0.5
58		4	>8	1	0.06	>8	0.125	0.25	>16	0.5
104		4	>8	1	0.06	>8	0.125	0.125	>16	0.25
103		8	>8	0.25	0.125	>8	0.5	0.25	>16	1
106		0.5	>8	0.25	0.125	>8	0.5	0.25	>16	0.5
113		4	>8	1	0.03	>8	0.125	0.5	>16	1
121		8	>8	2	0.06	>8	0.125	0.125	>16	0.25
140		8	>8	1	0.125	>8	0.25	0.125	>16	0.5
141		8	>8	2	0.125	>8	0.5	0.125	>16	0.25
143		4	>8	0.5	0.125	>8	0.5	0.5	>16	1
3	Environmental isolates from Gdansk and surroundings	>8	>8	>8	0.25	>8	0.25	2	>16	2
5		0.25	>8	0.25	0.06	>8	0.06	0.25	>16	0.25
8		0.25	>8	0.25	0.06	>8	0.06	0.25	>16	0.25

Table S3. The MIC values of ITR, POS and VOR after 20 transfers on Sabouraud agar with addition of POS, and then 5 transfers only on Sabouraud agar without any additions.

		**MIC values [mg/L] after 24 and 48 hours**								
**Tested Azoles**	Place of isolates origin	**ITR**			**POS**			**VOR**		
**Isolates**		**Initial value**	**After 20th**	**After 25th**	**Initial value**	**After 20th**	**After 25th**	**Initial value**	**After 20th**	**After 25th**
**2**	Clinical isolates from Wroclaw	0.25	>8	0.25	0.0312	>8	0.0312	0.25	>16	0.25
**13/2**		2	>8	2	0.5	>8	0.5	2	>16	2
**25**		0.5	>8	0.5	0.0312	>8	0.0312	0.25	>16	0.25
**29**		0.25	>8	0.25	0.0156	>8	0.0156	0.25	>16	0.25
**55**		>8	>8	>8	0.25	>8	0.25	2	>16	2
**72**		0.25	>8	0.25	0.0312	>8	0.0312	0.125	>16	0.125
**73**		0.125	>8	0.125	0.0156	>8	0.0156	0.25	>16	0.25
**79**	Clinical isolates from Warsaw	0.25	>8	0.25	0.0312	>8	0.0312	0.25	>16	0.25
**1**		0.5	>8	0.5	0.06	>8	0.06	0.5	>16	0.5
**5**		0.5	>8	0.5	0.03	>8	0.03	0.25	>16	0.25
**12**		0.5	>8	0.5	0.03	>8	0.03	0.25	>16	0.25
**13/3**		0.25	>8	0.25	0.03	>8	0.03	0.25	>16	0.25
**14**		0.5	>8	0.5	0.06	>8	0.06	0.25	>16	0.25
**15**		8	>8	8	0.5	>8	0.5	1	>16	1
**16**		0.5	>8	0.5	0.06	>8	0.06	0.25	>16	0.25
**20**		0.5	>8	0.5	0.03	>8	0.03	0.25	>16	0.25
**21**		>8	>8	>8	0.5	>8	0.5	2	>16	2
**22**		1	>8	1	0.06	>8	0.06	0.5	>16	0.5
**24**		8	>8	8	0.25	>8	0.25	1	>16	1
**37A**		0.5	>8	0.5	0.06	>8	0.06	0.25	>16	0.25
**132H**	Clinical isolates from Gdansk	0.5	>8	0.5	0.06	>8	0.06	0.25	>16	0.25
**1G**	Geese breeding	0.5	>8	0.5	0.06	>8	0.06	0.25	>16	0.25
**64**		4	>8	2	0.03	>8	0.125	0.5	>16	0.5
**67**		0.5	>8	0.25	0.06	>8	0.125	0.25	>16	0.25
**83**		8	>8	2	0.03	>8	0.06	0.25	>16	0.5
**84**		8	>8	1	0.03	>8	0.125	0.25	>16	0.5
**58**		4	>8	1	0.06	>8	0.5	0.25	>16	0.25
**104**		4	>8	2	0.06	>8	0.125	0.125	>16	0.25
**103**		8	>8	0.25	0.125	>8	0.5	0.25	>16	0.5
**106**		0.5	>8	0.5	0.125	>8	0.5	0.25	>16	0.5
**113**		4	>8	1	0.03	>8	0.06	0.5	>16	1
**121**		8	>8	2	0.06	>8	0.125	0.125	>16	0.5
**140**		8	>8	1	0.125	>8	0.5	0.125	>16	0.25
**141**		8	>8	1	0.125	>8	0.5	0.125	>16	0.5
**143**		4	>8	0.5	0.125	>8	0.25	0.5	>16	1
**3**	Environmental isolates from Gdansk and surroundings	>8	>8	>8	0.25	>8	0.25	2	>16	2
**5**		0.25	>8	0.25	0.06	>8	0.06	0.25	>16	0.25
**8**		0.25	>8	0.25	0.06	>8	0.06	0.25	>16	0.25

Table S4. The MIC values of ITR, POS and VOR after 20 transfers on Sabouraud agar with addition of VOR, and then 5 transfers only on Sabouraud agar without any additions.

		**MIC values [mg/L] after 24 and 48 hours**								
**Tested azoles**	Place of isolates origin	**ITR**			**POS**			**VOR**		
**Isolates**		**Initial value**	**After 20th**	**After 25th**	**Initial value**	**After 20th**	**After 25th**	**Initial value**	**After 20th**	**After 25th**
**2**	Clinical isolates from Wroclaw	0.25	>8	0.25	0.03	>8	0.03	0.25	>16	0.25
**13/2**		2	>8	2	0.5	>8	0.5	2	16	2
**25**		0.5	>8	0.5	0.03	>8	0.03	0.25	16	0.25
**29**		0.25	8	0.25	0.016	8	0.016	0.25	>16	0.25
**55**		>8	>8	>8	0.25	>8	0.25	2	>16	2
**72**		0.25	8	0.25	0.03	>8	0.03	0.125	>16	0.125
**73**		0.125	4	0.125	0.016	8	0.016	0.25	>16	0.25
**79**	Clinical isolates from Warsaw	0.25	8	0.25	0.03	4	0.03	0.25	>16	0.25
**1**		0.5	8	0.5	0.06	>8	0.06	0.5	>16	0.5
**5**		0.5	8	0.5	0.03	>8	0.03	0.25	>16	0.25
**12**		0.5	>8	0.5	0.03	>8	0.03	0.25	>16	0.25
**13/3**		0.25	>8	0.25	0.03	>8	0.03	0.25	>16	0.25
**14**		0.5	>8	0.5	0.06	8	0.06	0.25	>16	0.25
**15**		8	>8	8	0.5	>8	0.5	1	>16	1
**16**		0.5	8	0.5	0.06	>8	0.06	0.25	>16	0.25
**20**		0.5	>8	0.5	0.03	>8	0.03	0.25	>16	0.25
**21**		>8	>8	>8	0.5	>8	0.5	2	>16	2
**22**		1	>8	1	0.06	>8	0.06	0.5	>16	0.5
**24**		8	>8	8	0.25	>8	0.25	1	>16	1
**37A**		0.5	>8	0.5	0.06	>8	0.06	0.25	>16	0.25
**132H**	Clinical isolates from Gdansk	0.5	8	0.5	0.06	>8	0.06	0.25	>16	0.25
**1G**	Geese breeding	0.5	8	0.5	0.06	8	0.06	0.25	16	0.5
**64**		4	>8	2	0.03	>8	0.5	0.5	>16	1
**67**		0.5	>8	4	0.06	>8	0.5	0.25	>16	0.5
**83**		8	>8	4	0.03	>8	0.5	0.25	>16	1
**84**		8	>8	4	0.03	>8	0.25	0.25	>16	1
**58**		4	>8	2	0.06	>8	0.25	0.25	>16	0.5
**104**		4	>8	4	0.06	>8	0.125	0.125	>16	0.25
**103**		8	>8	0.5	0.125	>8	2	0.25	>16	1
**106**		0.5	>8	0.5	0.125	>8	2	0.25	>16	1
**113**		4	>8	2	0.03	>8	0.125	0.5	>16	1
**121**		8	>8	4	0.06	>8	0.5	0.125	>16	0.25
**140**		8	>8	8	0.125	>8	1	0.125	>16	0.125
**141**		8	>8	4	0.125	>8	1	0.125	>16	0.125
**143**		4	>8	4	0.125	>8	1	0.5	>16	1
**3**	Environmental isolates from Gdansk and surroundings	>8	>8	>8	0.25	>8	0.25	2	>16	2
**5**		0.25	4	0.25	0.06	>8	0.06	0.25	>16	0.25
**8**		0.25	4	0.25	0.06	>8	0.06	0.25	>16	0.25

Table S5. The alteration of *A. fumigatus* mycelium after exposition of POS, ITR and VOR.

<table>
<tr><th></th><th colspan="4">After 1th passage</th><th colspan="4">After 2^{sd} passage</th><th colspan="4">After $3\text{-}5^{th}$ passage</th><th>After $6\text{-}20^{th}$ passage</th></tr>
<tr><th>Isolate no.</th><th>POS</th><th>ITR</th><th>VOR</th><th>SAB</th><th>POS</th><th>ITR</th><th>VOR</th><th>SAB</th><th>POS</th><th>ITR</th><th>VOR</th><th>SAB</th><th>POS ITR VOR SAB</th></tr>
<tr><td>84</td><td rowspan="32">Mycelium was light creamy and grew deep in the medium. See Fig. S3</td><td rowspan="32">Mycelium was light creamy and grew deep in the medium. See Fig. S4</td><td rowspan="32">Classic, green A. fumigatus growth. See Fig. S2</td><td rowspan="6">Classic, green A. fumigatus growth</td><td rowspan="32" colspan="4">Classic, green A. fumigatus growth. See Fig. S5</td><td rowspan="21">Mycelium was green-brown-white. See Fig. S6</td><td rowspan="32" colspan="3">Classic, green A. fumigatus growth</td><td rowspan="32">Classic, green A. fumigatus growth</td></tr>
<tr><td>1</td></tr>
<tr><td>73</td></tr>
<tr><td>72</td></tr>
<tr><td>29</td></tr>
<tr><td>1</td></tr>
<tr><td>24</td><td>Growth deep in the medium, the darker color of mycelium from medium side. See Fig S1B</td></tr>
<tr><td>113</td><td rowspan="2">Classic A. fumigatus growth</td></tr>
<tr><td>140</td></tr>
<tr><td>21</td><td>Growth deep in the medium, the darker color of mycelium from medium side. See Fig S1B</td></tr>
<tr><td>37A</td><td rowspan="17">Classic, green A. fumigatus growth</td></tr>
<tr><td>121</td></tr>
<tr><td>141</td></tr>
<tr><td>2</td></tr>
<tr><td>22</td></tr>
<tr><td>14</td></tr>
<tr><td>67</td></tr>
<tr><td>143</td></tr>
<tr><td>85</td></tr>
<tr><td>20</td></tr>
<tr><td>25</td></tr>
<tr><td>79</td><td rowspan="2">White mycelium- (isolate no.79 and 103)</td></tr>
<tr><td>103</td></tr>
<tr><td>106</td><td rowspan="9">Classic, green A. fumigatus growth. See Fig. S5</td></tr>
<tr><td>13/3</td></tr>
<tr><td>12</td></tr>
<tr><td>5</td></tr>
<tr><td>13/2</td><td rowspan="2">Growth deep in the medium, the darker color of mycelium from medium side. See Fig S1B</td></tr>
<tr><td>15</td></tr>
<tr><td>132H</td><td rowspan="3">Classic, green A. fumigatus growth</td></tr>
<tr><td>83</td></tr>
<tr><td>64</td></tr>
</table>

**104**		
**16**		
**5**		
**8**		
**3**		
**55**	Growth deep in the medium, the darker color of mycelium from medium side. See Fig S1B	The brighter color of mycelium when compare with the classic mycelium, grew deep in the medium. See Fig. S7 and S8

In orange color *A. fumigatus* isolates from goose breeding are marked.

In blue color clinical *A. fumigatus* isolates are marked.

In green color environmental *A. fumigatus* isolates are marked

1st passage: on SAB and SAB supplemented by voriconazole

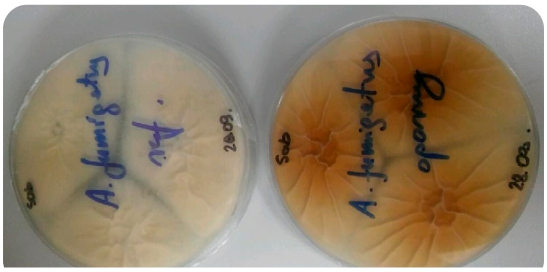

Figure S1. The growth of *A. fumigatus* isolate on SAB medium without any additions. A) *A. fumigatus* reference strain B) *A. fumigatus* isolates no. 13/2, 55, 15, 21, 24, 3 which grow deeper in the SAB medium.

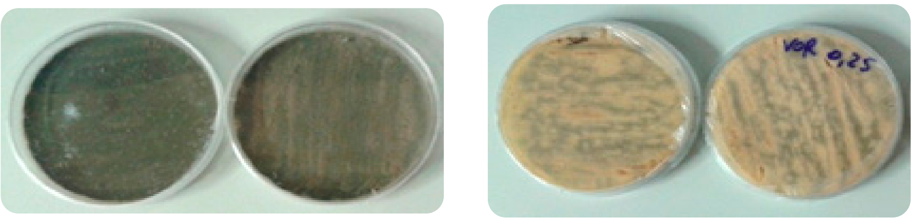

Figure S2. Colony morphology of isolate no. 2 cultured on A) SAB, B) SAB supplemented by 0,25 mg/L of VOR. The first picture was made from the mycelium side, the second from the medium side.

1st passage: on SAB and SAB supplemented by itraconazole

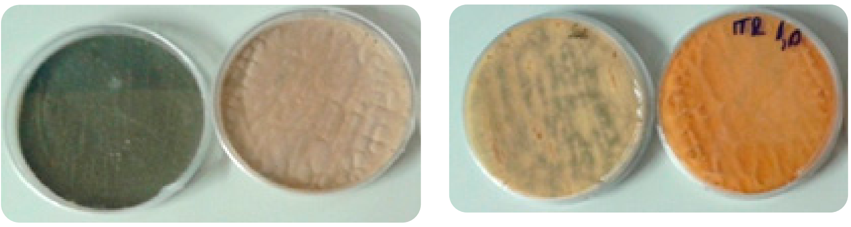

Figure S3. Colony morphology of isolate no. 2 cultured on A) SAB, B) SAB supplemented by 1 mg/L of ITR. The first picture was made from the mycelium side, the second from the medium side.

1st passage: on SAB and SAB supplemented by posaconazole

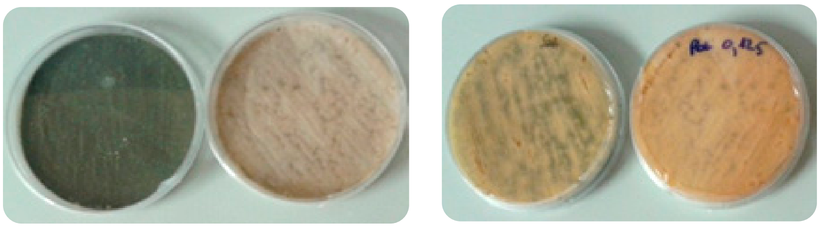

Figure S4. Colony morphology of isolate no. 2 cultured on A) SAB, B) SAB supplemented by 0,125 mg/L of POS. The first picture was made from the mycelium side, the second from the medium sideS4

2^{sd} passage: all tested isolates grew in classical manner as green colonies on SAB and SAB supplemented by azoles.

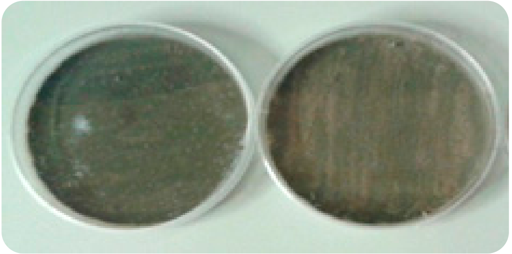

Colony morphology of isolate no. 2 cultured on A) SAB, B) SAB supplemented by 1 mg/L of ITR. Colony morphology of isolate no. 2 cultured on A) SAB, B) SAB supplemented by 1 mg/L of ITR.

3-5th passages: majority of isolates (instead of isolates no. 3 and 55) grew in classical manner as green colonies (see Fig, 5) on SAB and SAB supplemented by voriconazole and itraconazole. After 3-5th passages on SAB medium supplemented by posaconazole all isolates grew in different manner. The colonies were green-white-brown but in some cases only white.

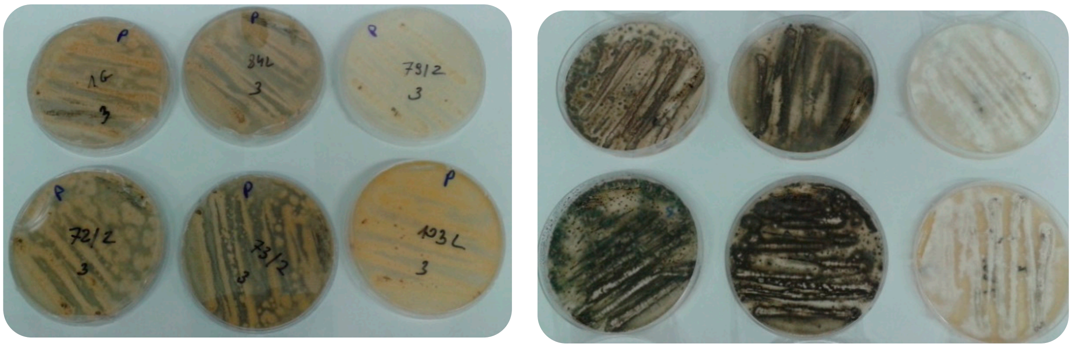

Figure S6. Colony morphology of isolates no. 1G, 84, 79, 72, 73 and 103 cultured on A) SAB, B) SAB supplemented by POS after 3-5th passages. The first picture was made from the mycelium side, the second from the medium side.

Morphology of isolates no. 3 and 55 were different after 3-5th passages on azoles when to compare with those isolates on SAB medium

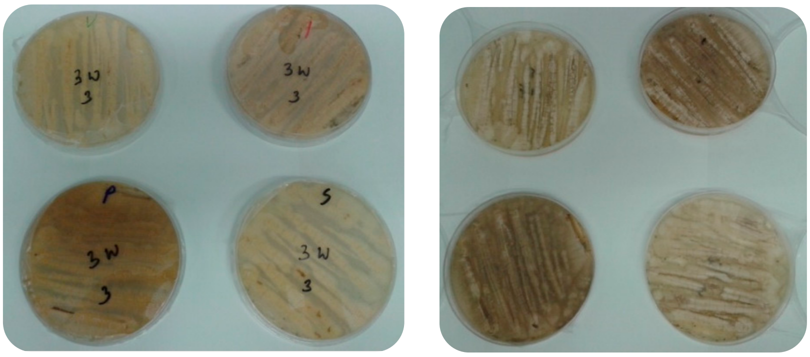

Figure S7. Colony morphology of isolate no. 3 cultured on SAB supplemented by VOR, ITR, POS and only SAB after 3-5th passages. The first picture was made from the mycelium side, the second from the medium side.

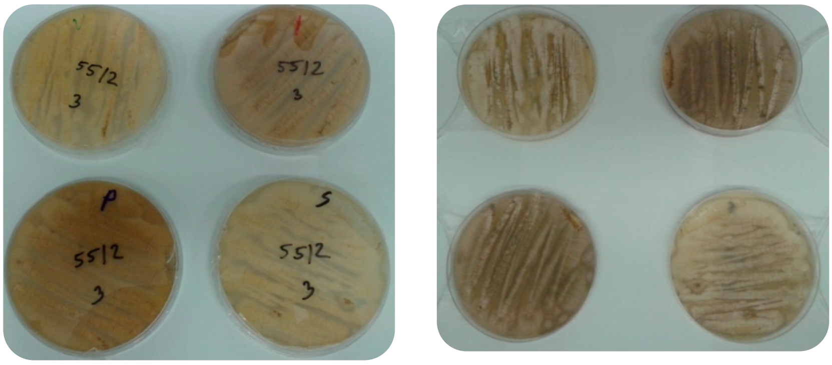

Figure S8. Colony morphology of isolate no. 55 cultured on SAB supplemented by VOR, ITR, POS and only SAB after 3-5th passages. The first picture was made from the mycelium side, the second from the medium side.

Table S6. The list of primer sequence used in this study.

Gene	Primer sequence (5′ –> 3′)	Reference
mdr1	GCTCTTCCCTTGTTCACAATTC	This study
	CGGCAATACCGAGATACACA	
mdr2	TTTAGCTCCACCGGGTTTG	[25]
	TCGAAAGACCGAACATGCTTGA	
mdr3	TCTGATGGCGGTCATCACT	[25]
	ATATCCATCCCCCAGGC	
atrf	AGAGAAATCGGACAACTGCTGA	[25]
	CCTCGTCGCAGATAGTCTTGTA	
cyp51A	TGCAGAGAAAAGTATGGCGA	[25]
	CGCATTGACATCCTTGAGC	
cyp51B	AGCAGAAGAAGTTCGTCAAATAC	[25]
	TCGAAGACGCCCTTGTG	
**β-tubulin**	TTCCCCCGTCTCCACTTCTTCATG	[26]
	GACGAGATCGTTCATGTTGAACTC	

Table S7. Raw data of cycle quantification values and standard deviation of tested genes. Continuation of this table on the next page.

**Cycle quantification values (Cq) ± standard deviation (SD) of following genes after:**							
**Culturing on SAB**							
**Isolate**	***β-tub***	***mdr1***	***mdr2***	***mdr3***	***atrF***	***cyp51A***	***cyp51B***
**13/2**	**28.35** ± 0.05	**22.56** ± 0.27	**31.62** ± 0.16	**31.48** ± 0.03	**25.64** ± 0.13	**27.65** ± 0.05	**31.19** ± 0.03
**3**	**28.64** ± 0.27	**25.17** ± 0.26	**28.41** ± 0.00	**25.87** ± 0.06	**21.52** ± 0.13	**22.87** ± 0.10	**31.40** ± 0.04
**2**	**27.36** ± 0.03	**23.79** ± 0.04	**31.74** ± 0.17	**33.76** ± 0.05	**27.61** ± 0.09	**24.88** ± 0.07	**32.97** ± 0.47
**67**	**31.25** ± 0.03	**22.57** ± 0.01	**31.30** ± 0.27	**32.36** ± 0.07	**27.56** ± 0.19	**27.25** ± 0.05	**34.15** ± 0.14

microorganisms

MDPI

Table S7 continuation. Raw data of cycle quantification values and standard deviation of tested genes.

Cycle quantification values (Cq) ± standard deviation (SD) of following genes after:														
**20-times culturing with ITR**								**20-times culturing with ITR + 5-times on SAB**						
**Isolate**	***β-tub***	***mdr1***	***mdr2***	***mdr3***	***atrF***	***cyp51A***	***cyp51B***	***B-tub***	***mdr1***	***mdr2***	***mdr3***	***atrF***	***cyp51A***	***cyp51B***
**13/2**	**33.62** ± 0.05	**27.59** ±0.21	**31.52** ± 0.16	**32.25** ± 0.36	**23.63** ± 0.12	**28.02** ± 0.06	**33.59** ± 0.09	**31.02** ± 0.50	**25.79** ± 0.14	**29.53** ± 0.07	**28.86** ± 0.08	**19.43** ± 0.21	**25.01** ± 0.01	**33.28** ± 0.34
**3**	**33.64** ± 0.2	**27.60** ±0.12	**31.57** ± 0.06	**30.81** ± 0.05	**24.66** ± 0.17	**27.69** ± 0.27	**35.15** ± 0.21	**32.55** ± 0.03	**25.47** ± 0.18	**33.60** ± 0.26	**35.09** ± 0.24	**27.54** ± 0.04	**30.57** ± 0.21	**33.82** ± 0.18
**2**	**33.62** ± 0.44	**27.60** ±0.04	**35.68** ± 1.69	**34.01** ± 0.47	**27.60** ± 0.13	**30.29** ± 0.07	**33.16** ± 0.15	**32.56** ± 0.35	**24.78** ± 0.06	**31.58** ± 0.22	**32.08** ± 0.71	**22.43** ± 0.35	**26.44** ± 0.15	**34.58** ± 0.44
**67**	**32.68 ±0.06**	**27.58 ± 0.04**	**34.27** ± 0.84	**33.84** ± 0.11	**28.17** ± 0.35	**30.55** ± 0.34	**32.96** ± 0.65	**33.01** ± 0.83	**25.55** ± 0.12	**32.45** ± 0.58	**31.12** ± 0.40	**26.80** ± 0.13	**29.54** ± 0.02	**33.88** ± 0.06
**20-times culturing with VOR**								**20-times culturing with VOR + 5-times on SAB**						
**Isolate**	***β-tub***	***mdr1***	***mdr2***	***mdr3***	***atrF***	***cyp51A***	***cyp51B***	***B-tub***	***mdr1***	***mdr2***	***mdr3***	***atrF***	***cyp51A***	***cyp51B***
**13/2**	**33.35** ± 0.64	**27.45** ± 0.04	**37.75** ± 0.22	**35.15** ± 0.50	**29.11** ± 0.80	**31.66** ± 0.06	**33.24** ± 0.47	**32.39** ± 0.44	**24.88** ± 0.44	**35.94** ± 0.00	**32.56** ± 1.36	**28.76** ± 0.25	**31.67** ± 0.20	**33.46** ± 0.30
**3**	**30.70** ± 0.32	**26.77** ± 0.03	**28.45** ± 0.64	**28.85** ± 0.66	**21.87** ± 0.12	**24.68** ± 0.22	**32.69** ± 0.38	**32.71** ± 0.16	**25.49** ± 0.32	**33.80** ± 1.14	**34.07** ± 0.50	**28.08** ± 0.57	**32.23** ± 0.32	**33.52** ± 0.57
**2**	**31.67** ± 0.24	**27.42** ± 0.13	**31.47** ± 0.03	**31.19** ± 0.72	**24.20** ± 0.30	**27.85** ± 0.12	**33.35** ± 0.04	**33.03** ± 0.18	**24.63** ± 0.37	**35.86** ± 0.04	**33.89** ± 0.13	**25.58** ± 0.10	**29.38** ± 0.08	**34.18** ± 0.11
**67**	**32.98** ± 0.74	**26.62** ± 0.28	**31.52** ± 0.43	**36.52** ± 0.30	**27.92** ± 0.07	**32.73** ± 0.35	**33.22** ± 0.25	**31.74** ± 0.35	**24.61** ± 0.26	**33.57** ± 1.12	**34.12** ± 0.82	**27.70** ± 0.29	**29.52** ± 0.14	**33.46** ± 0.17
**20-times culturing with POS**								**20-times culturing with POS + 5-times on SAB**						
**Isolate**	***β-tub***	***mdr1***	***mdr2***	***mdr3***	***atrF***	***cyp51A***	***cyp51B***	***B-tub***	***mdr1***	***mdr2***	***mdr3***	***atrF***	***cyp51A***	***cyp51B***
**13/2**	**33.40** ± 0.04	**26.90** ± 1.29	**32.75** ± 0.20	**34.38** ± 0.06	**27.67** ± 0.38	**29.36** ± 0.21	**33.81** ± 0.25	**32.05** ± 0.30	**26.05** ± 0.38	**33.75** ± 0.12	**33.66** ± 0.31	**27.21** ± 0.39	**31.71** ± 0.96	**32.66** ± 0.15
**3**	**32.36** ± 0.73	**26.05** ± 0.48	**30.58** ± 0.71	**31.66** ± 0.35	**24.36** ± 0.74	**28.63** ± 0.26	**33.73** ± 0.13	**33.04** ± 0.53	**24.92** ± 0.41	**32.11** ± 1.20	**33.14** ± 0.44	**25.83** ± 0.00	**29.19** ± 0.21	**34.58** ± 0.16
**2**	**33.23** ± 0.49	**26.53** ± 0.17	**32.33** ± 0.46	**31.85** ± 0.21	**23.84** ± 1.37	**26.85** ± 0.12	**33.74** ± 0.71	**31.64** ± 0.11	**24.51** ± 0.86	**32.77** ± 1.94	**32.86** ± 0.72	**23.82** ± 0.08	**27.28** ± 0.37	**35.36** ± 0.12
**67**	**34.65** ± 0.33	**26.62** ± 0.09	**33.97** ± 0.81	**32.06** ± 0.82	**30.67** ± 0.27	**33.38** ± 0.39	**33.15** ± 0.13	**32.85** ± 0.10	**24.12** ± 0.59	**32.93** ± 1.53	**31.87** ± 1.32	**27.62** ± 0.15	**31.20** ± 0.18	**34.17** ± 0.17
